# A new carboxypeptidase from *Aspergillus niger* with good thermostability, pH stability and broad substrate specificity

**DOI:** 10.1038/s41598-021-98003-x

**Published:** 2021-09-21

**Authors:** Peng Song, Wei Xu, Yang Zhang, Fei Wang, Xiuling Zhou, Haiying Shi, Wei Feng

**Affiliations:** School of Life Sciences, Liaocheng University, Liaocheng, 252000 China

**Keywords:** Biotechnology, Molecular biology

## Abstract

A new serine carboxypeptidase gene, *capA*, was identified in *Aspergillus niger* CBS 513.88 by reading genomic information and performing sequence alignment, and the gene was cloned and expressed in *Pichia pastoris* GS115. In a shake flask, the enzyme activity of the recombinant strain GS115 (pPIC9K-*capA*) reached 209.3 U mg^−1^. The optimal temperature and pH for enzyme activity were determined to be 45 °C and 6.0, respectively. After incubation at 40–50 °C or at pH 4.0–8.0 for 1 h, the enzyme retained more than 80% or 60% of its initial activity. The presence of 1–10 mmol L^−1^ Mg^2+^ enhanced the activity of CapA, whereas 1–10 mmol L^−1^ Cu^2+^, Fe^2+^, or Co^2+^, 10 mmol L^−1^ Mn^2+^, or 1–10 mmol L^−1^ phenylmethylsulfonyl fluoride (PMSF) significantly inhibited its activity. CapA had a broad substrate specificity and preferred the hydrophobic amino acids Leu and Lys at the C terminus of proteins, and *N*-benzyloxycarbonyl-l-phenylalanyl-l-leucine (Cbz-Phe-Leu) was the optimal substrate, for which CapA exhibited *K*_m_ 0.063 mmol L^−1^ and *k*_cat_*/K*_m_ 186.35 mmol L^−1^ s^−1^. The good thermostability, pH stability and hydrolysis characteristics of CapA provide a solid foundation for application in the food and biotechnology fields.

## Introduction

Carboxypeptidases are exoproteases that hydrolyze the C-terminal peptide bonds of proteins or polypeptides and release free amino acids individually. Carboxypeptidases are divided into serine carboxypeptidases (EC 3.4.16.), metal carboxypeptidases (EC 3.4.17.), and cysteine carboxypeptidases (EC 3.4.18.) based on the differences in their catalytic mechanisms^1^. Carboxypeptidases are commonly used in the food industry to produce amino acids^2^, prepare oligopeptides^3,4^, debitter protein hydrolysates and enhance flavor^5,6^. Some special carboxypeptidases are also used to cleave specific polypeptides^7^ and amino acid sequences within polypeptides^8,9^ in the field of biotechnology.

Carboxypeptidases are found in animals, plants, fungi and bacteria^10^, but the content of carboxypeptidase in animals and plants is low, the composition is complex, and the extraction cost is high. Microbial fermentation is the main method of obtaining carboxypeptidases. At present, carboxypeptidases from various sources, mainly microbial carboxypeptidases and especially fungal carboxypeptidases, have been subjected to taxonomic study^11^, and some have been cloned and expressed^12–14^. Among these identified carboxypeptidases, two kinds of carboxypeptidases originating from *A. niger* have been cloned and expressed: CPD-I (PepF) and CPD-II (PepG)^15^. In addition, a patent (patent US 5939305)^16^ reported a gene from *A. niger* encoding a carboxypeptidase**.**

The genomic sequence of *A. niger* CBS 513.88 was analyzed and published in 2007. The *A. niger* protein database shows that there are 15 putative carboxypeptidase-encoding genes^17^; except for CPD-I and CPD-II^15^, the rest have undetermined functions. In this paper, one predicted gene encoding a serine carboxypeptidase was first cloned and expressed in *Pichia pastoris*, and the enzymatic properties of the recombinant enzyme were systematically analyzed. This research has laid a good foundation for further exploring the application value of this enzyme.

## Materials and methods

### Materials and reagents

*Aspergillus niger* CGMCC 3.7193 was stored in the China Common Microbe Culture Collection Management Center (CGMCC); *E. coli* JM109 was obtained from Promega (Madison, WI); *P. pastoris* GS115 and the pPIC9K plasmid were obtained from Invitrogen (San Diego, CA, USA); all were stored at − 80 °C. *A. niger* and *E. coli* were cultured on potato dextrose agar (PDA) medium (2% potato, 2% dextrose and 2% agar) and Luria–Bertani (LB) medium (10% tryptone, 5% yeast extract and 10% NaCl), respectively. *P. pastoris* was cultured in yeast extract peptone dextrose (YPD) medium (1% yeast extract, 2% peptone and 2% dextrose), minimal dextrose (MD) medium (1.34% yeast nitrogen base without amino acids, 2% dextrose and 0.00004% biotin), buffered glycerol-complex (BMGY) medium (1% yeast extract, 2% peptone, 1.34% yeast nitrogen base without amino acids, 100 mmol L^−1^ potassium phosphate at pH 6.0, and 1% glycerol) and buffered methanol-complex (BMMY) medium (1% yeast extract, 2% peptone, 1.34% yeast nitrogen base without amino acids, 100 mmol L^−1^ potassium phosphate at pH 6.0, and 0.5% methol). The preparation and culture methods were performed according to the Pichia Expression Kit (Version M) provided by Invitrogen. The polymerase chain reaction (PCR) primers for CapA were F: 5′-GTAGTCCTCCAGCCAGAGGAACCATC-3′ and R: 5′-TGCTCTAGATCACTCAGTAAACGATGCCCCG-3′; the restriction enzyme sites are underlined, and these primers were generated by Sangon Biotech Co., Ltd. (Shanghai). The restriction endonuclease, *Pyrobest* DNA polymerase and T4 DNA ligase were purchased from the TaKaRa company; the Plasmid Extraction Kit and DNA Purification and Recovery Kit were purchased from Beijing Zoman Biotechnology Co., Ltd; the RNAqueous™-Micro Total RNA Isolation Kit and SuperScript III First-Strand Synthesis System were purchased from Invitrogen; and the following chemical substrates were synthesized by Ontores Biotech Co: CBZ-ala-Arg, CBZ-Pro-Gly, CBZ-Ala-Lys, CBZ-Gly-Ala, CBZ-Ala-Glu and CBZ-Phe-Leu.

### Methods

#### Gene cloning and recombinant *P. pastoris* construction

The total RNA was extracted from *A. niger*; first-strand cDNA was synthesized by reverse transcription and used as a template for PCR amplification with the synthesized primer; and the subsequent PCR product purification, enzyme digestion, ligation, *E. coli* JM109 transformation, and positive clone screening were performed according to routine laboratory methods^18^. Recombination plasmid linearization and electronic transformation of *P. pastoris* GS115 were conducted, and recombinant *P. pastoris* GS115 (pPIC-*capA*) was screened according to the methods provided by the Pichia Expression Kit.

#### Induction of expression and purification of recombinant enzyme

Recombinant *P. pastoris* GS115 (pPIC-CapA) was purified on YPD plates. Single colonies were selected, inoculated into YPD liquid medium (25 mL) and cultured at 30 °C and 200 r min^−1^ for 18 h to 20 h. Then, 1% of the inoculation was transferred into BMGY medium (25 mL). The bacteria were cultured at 30 °C and 200 r min^−1^ for 16 h-18 h until the cells reached the logarithmic stage of growth (absorbance 600 nm reached 2.0–6.0). Then, the bacteria were centrifuged at 8000 r min^−1^ for 5 min for collection, and the bacteria were suspended in the proper volume of BMMY medium and cultured until the absorbance value at 600 nm reached 1.0. For induction, the bacteria were cultured at 30 °C and 200 r min^−1^. Methanol was added every 24 h until the final concentration reached 0.5%. The fermentation was maintained for 120 h and then ended. The supernatant of the fermentation broth was centrifuged at 4 °C and 8000 r min^−1^ to collect the crude enzyme broth.

The crude enzyme solution was precipitated by 30–70% ammonium sulfate and dialyzed by using a dialysis bag with a molecular weight cutoff of 50 kDa. After dialysis, the collections were further loaded onto a Q-Sepharose Fast Flow anion-exchange column (GE Healthcare Bio-Sciences). The protein was eluted by a linear gradient using buffer A (20 mmol L^−1^ sodium phosphate buffer, pH 6.0) and buffer B (20 mmol L^−1^ sodium phosphate pH 6.0, 0.5 m NaCl). The active fractions were pooled and dialyzed against 0.1 mol L^−1^ disodium hydrogen phosphate-citric acid buffer (pH 6.0) and then used for enzyme kinetics studies. The protein concentration was determined using the Bradford assay^19^ with bovine serum albumin Fractionation V (Sigma) as the reference standard.

#### Determination of recombinant carboxypeptidase activity

Carboxypeptidase activity was determined by referring to the method described by Morita et al.^3^, and a simple optimization was performed. Using CBZ-Phe-Leu as the substrate, a 1 mmol L^−1^ substrate solution was prepared with a pH 6.0 disodium hydrogen phosphate and citric acid buffer solution (0.1 mol L^−1^). A total of 450 μL of substrate and 50 μL of the suitably diluted enzyme solution were mixed and allowed to reacted at 37 °C for 60 min. A 500-μL aliquot of a 0.5% indanone solution was immediately added; the mixture was heated in a water bath at 100 °C for 15 min and then cooled by tap water for 5 min. The absorbance value A570 was determined by spectrophotometry (SP-2012UV spectrophotometer: Shanghai Spectral Instrument Co., Ltd.). Standard tyrosine solutions of different concentrations were prepared and allowed to react with ninhydrin under the same conditions. The standard curve was generated.

The definition of the enzyme activity is as follows: the unit of enzyme activity (U) is the amount of enzyme that hydrolyzes the substrate to generate 1 μg tyrosine at 37 °C for 1 min.

#### Analysis of the enzymatic properties of recombinant carboxypeptidase

##### Determination of optimum temperature and temperature stability

The activity levels of carboxypeptidase were determined at 30–70 °C and pH 6.0, and the optimal reaction temperature of the enzyme was determined.

The enzyme solution was incubated at 30–70 °C for 0.5, 1, 1.5 and 2 h. The enzyme activity was measured according to the method described in “Determination of recombinant carboxypeptidase activity”. The enzyme solution without heat treatment was used as a control (the enzyme activity was 100%) to calculate the relative enzyme activity and investigate the thermal stability of carboxypeptidase.

##### Determination of the optimum pH and pH stability

The activity values of carboxypeptidase at pH 4.0–8.0 and 45 °C were measured to determine the optimal pH of the enzyme.

The enzyme solution was incubated in a pH 4.0–8.0 buffer solution for 1 h (45 °C), and the enzyme activity was determined according to the method described in “Determination of recombinant carboxypeptidase activity”. The maximum value of the enzyme activity was 100%, and the pH stability of carboxypeptidase was investigated. The buffer used was 0.1 mol L^−1^ disodium hydrogen phosphate-citric acid buffer at pH range from 4.0 to 8.0.

##### Influence of metal ions and chemical reagents on enzyme activity

Metal ions and chemical reagents at final concentrations of 1 mmol L^−1^ were added to the carboxypeptidase and substrate reaction systems. The enzyme activity was determined according to the method described in “Determination of recombinant carboxypeptidase activity”. The enzyme activity in the system without metal ions and chemical reagents was 100% (the control was measured in the presence of 10 mmol L^−1^ EDTA), and the relative enzyme activity in the presence of the metal ions and chemical reagents was calculated.

##### Substrate specificity analysis

Carboxypeptidase was reacted with 1 mmol L^−1^ CBZ-Ala-Arg, CBZ-Pro-Gly, CBZ-Ala-Lys, CBZ-Gly-Ala, CBZ-Ala-Glu or CBZ-Phe-Leu, and the enzyme activity was determined according to the method described in “Determination of recombinant carboxypeptidase activity”. When CBZ-Phe-Leu was used as the substrate, the enzyme activity in the assay was 100%. The relative enzyme activity value of other substrates reacting with carboxypeptidase was calculated.

##### Determination of kinetic parameters

Enzyme assays were performed using optimal conditions with different concentrations of substrates (0.05–2 mmol L^−1^). The kinetic parameters *K*_m_ and *V*_max_ were evaluated from Lineweaver–Burk plots^20^ of the initial velocity at a substrate concentration of 0.05–2 mmol L^−1^, and *k*_cat_ was the value of *V*_max_ divided by *E* (enzyme concentration).

#### Bioinformatics analysis

NCBI (https://www.ncbi.nlm.nih.gov/genbank/) was used to identify the serine carboxypeptidase amino acid sequences from *A. niger* and other sources; Clustal X2 and Biodit were used for sequence alignment analysis. The phylogenetic relationship was analyzed with Mega 5.0 by the neighbor-joining method^21^. The signal peptide was predicted using SignalP 5.0 Server (http://www.cbs.dtu.dk/services/SignalP/). The *N*-glycosylation sites were analyzed by the NetNGlyc 1.0 server (www.cbs.dtu.dk/services/NetNGlyc/).

#### Statistical analysis

The data presented are the means and standard error of three independent experiments. Analyses of variance were carried out using SAS version 8.02 (SAS Institute Inc., Cary, NC, USA). Comparisons were considered significantly different if *P* < 0.05.

## Results and discussion

### Cloning and sequence analysis of the carboxypeptidase gene

The *A. niger* CBS 513.88 genome (EMBL AM270980-AM270998) has been sequenced^17^, and a novel suspected carboxypeptidase (CapA) gene has been identified by analyzing its genome information and BLAST. The cDNA of *A. niger* F0510 was extracted as a template, and the gene was successfully amplified using CapA-F and CapA-R as primers. The recombinant plasmid pPIC-CapA was constructed. Sequencing confirmed that CapA had an intact open reading frame (ORF), and the nucleotide sequence was identical to the published sequence (Locus tag: ANI_1_238044) of the *A. niger* CBS 513.88 genome. The serine carboxypeptidase gene contains 1479 bp and encodes 492 amino acids, and analysis of the predicted protein sequence suggested that CapA has a signal peptide of 19 amino acids in the N terminus.

**Figure 1:**
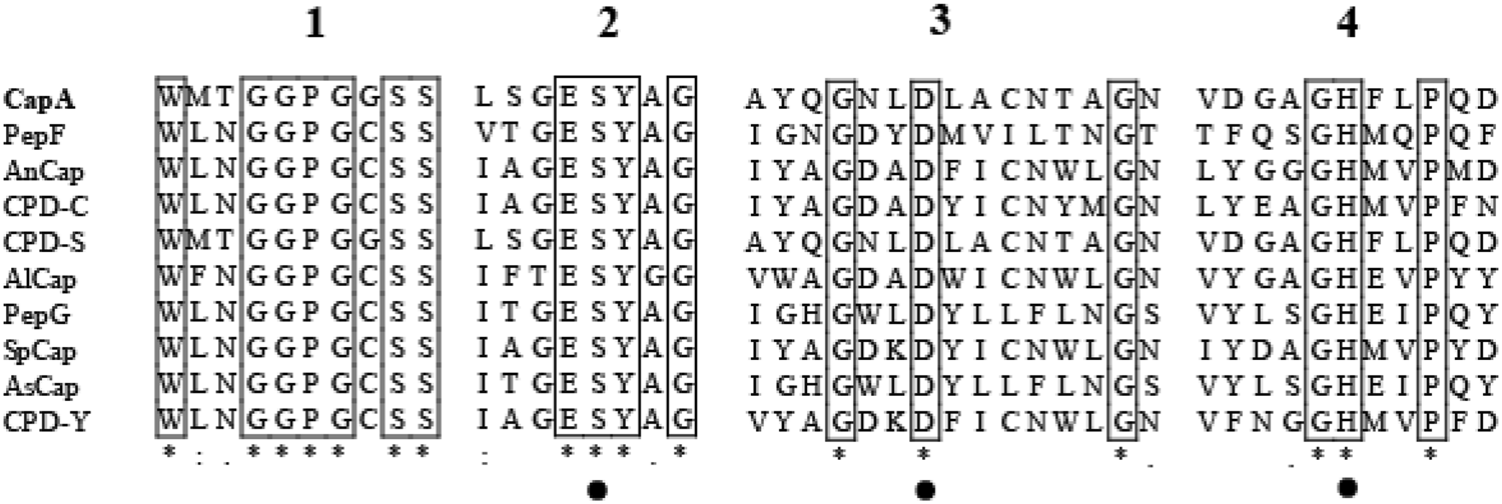
Multiple sequence alignment of serine carboxypeptidases from different microorganisms. (*): conserved amino acid; (:): conservative replacement; (.): semi-conservative substitution; and the amino acid residues of the active sites are marked with black dots. The four conservative domains (1, 2, 3, and 4) are indicated above.

Clustal X2 was used to analyze and compare the amino acid sequences of serine carboxypeptidases from different sources, which are listed and summarized in Fig. 1. There are four conserved domains involved in the substrate binding and catalysis of CapA, among which domain 1 is the conserved substrate binding domain, and domains 2–4 are the conserved catalytic domains, which include the conserved triplet of serine (S), aspartic acid (D) and histidine (H)^3,10^. A conserved G-X-S-X sequence (located in domain 1) spatially includes the active serine residues^22^. There is a conserved glutamate (Glu) in front of the catalytic serine residue (located in domain 2), which is thought to be the reason serine carboxypeptidase performs the best catalysis under acidic conditions^22^. Similar to other carboxypeptidases, CapA belongs to the S10 family of SC carboxypeptidases^23^.

**Figure 2:**
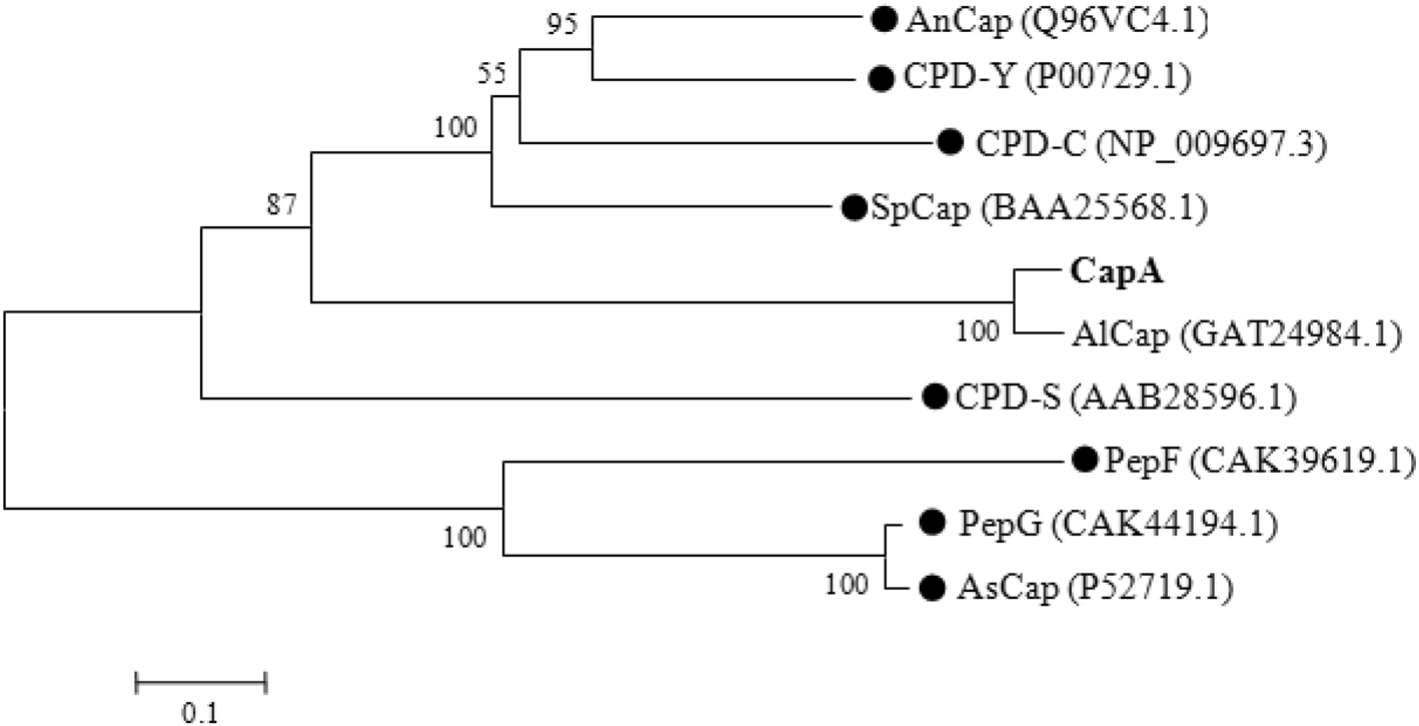
Phylogenetic tree describing the genetic distances among various serine carboxypeptidases from different microorganisms. The serine carboxypeptidase in this research is shown in bold. The length of the line segment is the distance calculated by MEGA 5.0. The number on the branch node represents the bootstrap percentage. Values less than 50% are not shown. The black dots indicate that the carboxypeptidase has been reported in the literature. The entry number of the enzyme protein in GenBank is indicated in brackets. AnCap^24^ is from *A. nidulans*, CPD-Y^25^ and CPD-C^26^ is from *S. cerevisiae*, SpCap^27^ is from *S. pombe*, AlCap is from *A. luchuensis*. CPD-S^28^ is derived from *P. janthinellum*, PepF and PepG^15^ from *A. niger*, and AsCap^29^ from *A. saitoi*.

CapA was further compared with the amino acid sequences reported for serine carboxypeptidases using MEGA 5.0, and a phylogenetic tree was constructed, as shown in Fig. 2. The phylogenetic tree reflects the genetic distance between 10 different serine carboxypeptidases, and a short genetic distance and clustering indicate a close genetic relationship. The amino acid sequence similarity between CapA and other serine carboxypeptidases ranges from 15.76% (SpCap) to 93.09% (AlCap), with an average of 30.45%.

### Expression and induction of carboxypeptidase CapA

**Figure 3:**
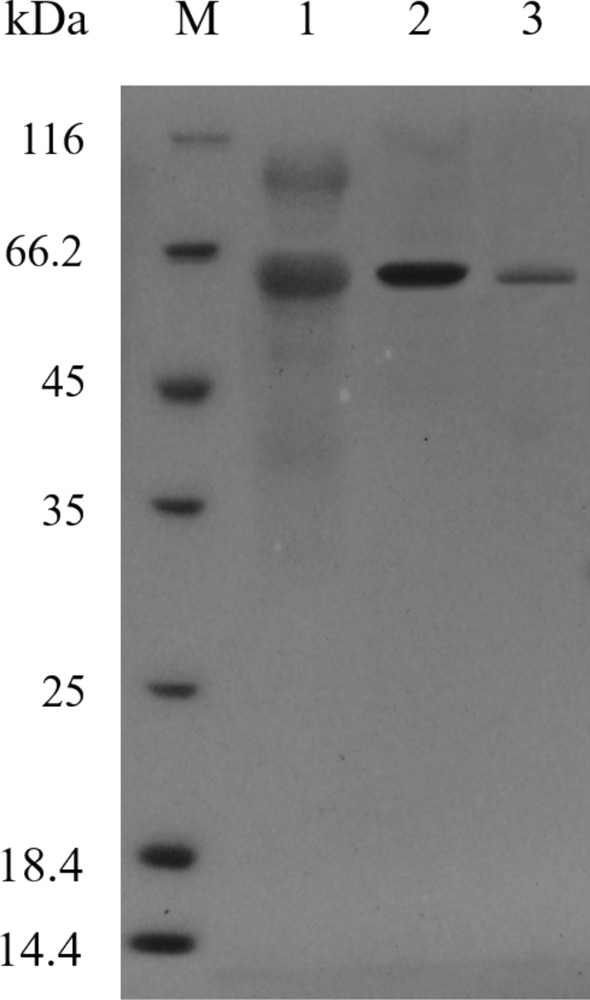
SDS-PAGE analysis of the purification of the recombinant enzyme CapA. M: Protein molecular weight standard; 1: crude enzyme solution; 2: purified CapA by Ammonium sulfate precipitation and dialysis; 3: purified CapA by Q-Sepharose anion-exchange column chromatography.

**Figure 4:**
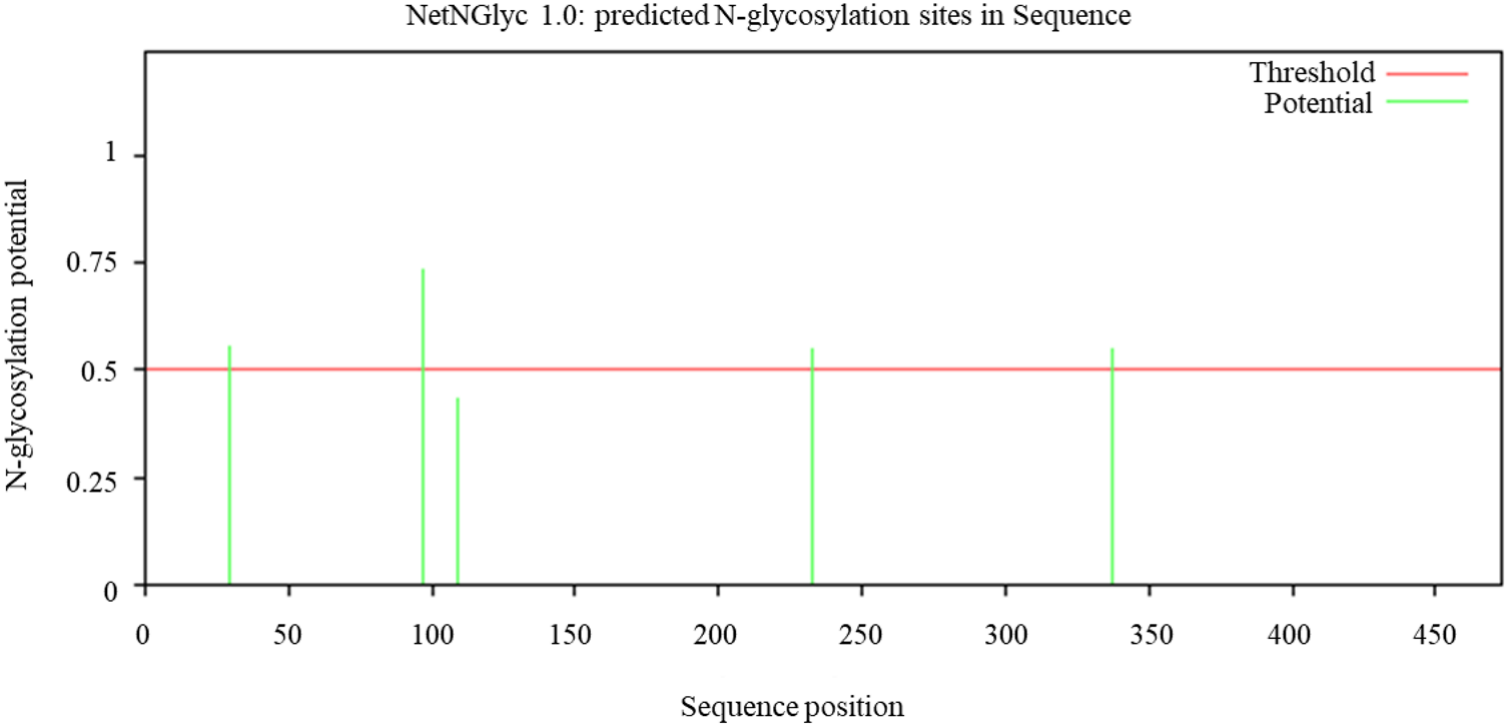
NetNGlyc 1.0 identification of several potential *N*-glycosylation sites in carboxypeptidase CapA. The potential *N*-glycosylation sites are those that are above the threshold (0.5), with the highest peaks indicating the greatest potential for glycosylation.

The recombinant plasmid pPIC-CapA was linearized with the restriction enzyme *Sac* I and electrically transformed into *P. pastoris* GS115. His^+^ transformants growing on YPD agar plates with G418 at two concentrations (0.5 and 2.0 mg mL^−1^) after previously growing on MD plates at 30 °C for 3 d were selected. A single colony from the YPD plate with 2.0 mg mL^−1^ G418 was inoculated into BMGY to an absorbance value of 4.0–6.0 at 600 nm and transferred into BMMY for expression and induction of the recombinant protein. The maximum enzyme activity of the crude enzyme reached 209.3 U mg^−1^ after 120 h of culture with methanol in a shaking flask. After successive purification of the crude CapA enzyme solution by salting out, dialysis and ion exchange chromatography, the specific activity of purified CapA reached 495.7 U mg^−1^ (Table 1). Sodium dodecyl sulfate–polyacrylamide gel electrophoresis (SDS-PAGE) (Fig. 3) analysis showed that the molecular weight of CapA was approximately 60.0 kDa, which was slightly higher than the theoretical molecular weight of 52.7 kDa; this difference was speculated to be caused by *N*-glycosylation. *N*-Glycosylation is one of the most common modifications occurring in the synthesis of proteins in *P. pastoris*^30^. This speculation was supported by the prediction of *N*-glycosylation sites by the NetNGlyc 1.0 Server: Asn29, Asn97, Asn233 and Asn337 were potential *N*-glycosylation sites in the mature peptide of CapA based on the locations in consensus NxT/S sequences^31^ (Fig. 4).

**Table 1:** Purification scheme of the CapA.

Purification steps	Total protein (mg)	Total activity (U)	Specific activity (U mg^−1^)	Enrichment (fold)	Yield (%)
Crude extract	124.5	26,057.9	209.3	1	100
Ammonium sulfate precipitation and dialysis	44.6	17,282.5	387.5	1.9	66
Q-Sepharose	2.8	1388	495.7	2.4	5

### Enzymatic properties of carboxypeptidase

#### Optimal temperature and temperature stability

**Figure 5:**
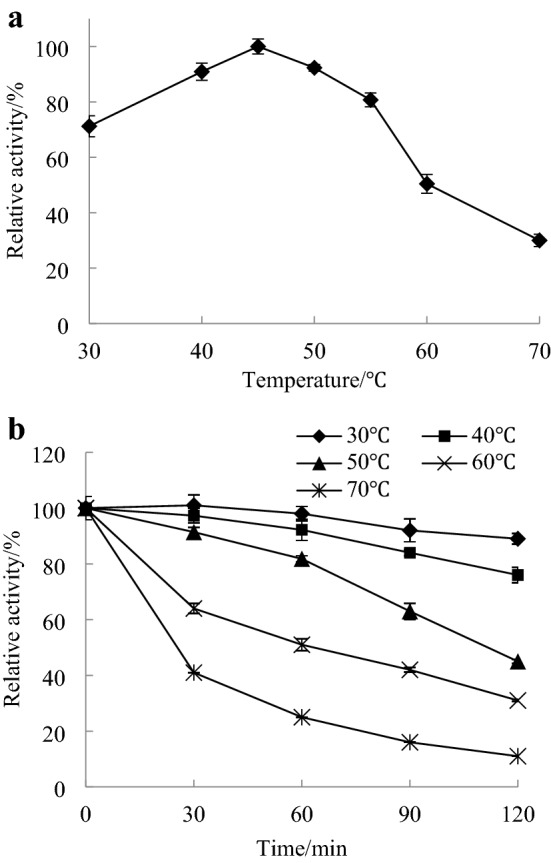
Effect of temperature on the activity (**a**) and stability (**b**) of CapA.

Carboxypeptidase activity was detected in the range of 30–70 °C, and the results are shown in Fig. 5a. The optimal reaction temperature of CapA was 45 °C, and the relative activity could be maintained at more than 70% at temperatures of 30–55 °C. The thermal stability study (Fig. 5b) showed that after incubation at 30–50 °C for 1 h, the enzyme activity of CapA remained greater than 80%, and after incubation at 60 °C and 70 °C for 2 h, the enzyme activity remained greater than 30% and 10%, respectively.

The optimal reaction temperature and thermal stability of CapA were significantly higher than those of *A. oryzae* carboxypeptidase^3^, which had an optimal reaction temperature of 30 °C and less than 10% enzyme activity after 30 min incubation at 60 °C. The optimal values of CapA were also higher than the optimal temperature (30 °C) and thermal stability of *S. cerevisiae*-derived recombinant carboxypeptidase Y (after 60 °C incubation for 1 h, the enzyme activity was almost undetectable)^25^. CapA has better heat resistance than the other carboxypeptidases, resulting in the advantages of a simplified process, improved the efficiency and reduced the cost in application^32^.

#### Optimal pH and pH stability

**Figure 6:**
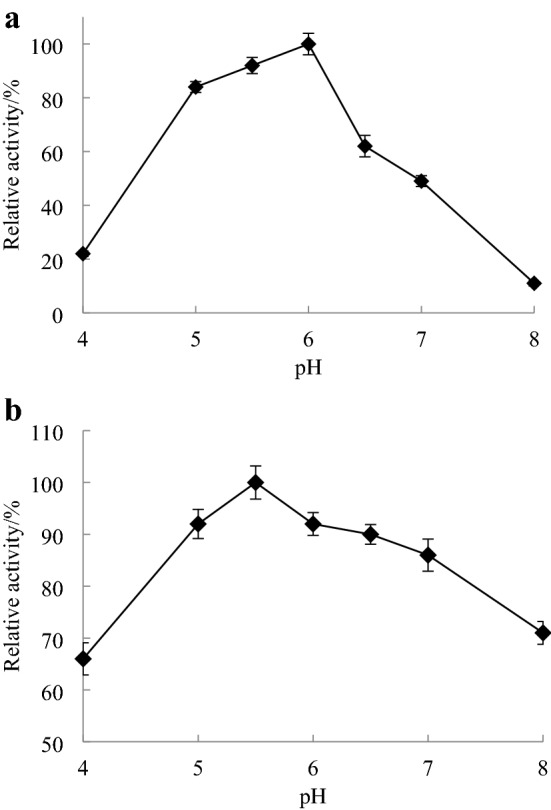
Effect of pH on the activity (**a**) and stability (**b**) of CapA.

Carboxypeptidase activity was detected in the pH range of 4.0–8.0, and the results are shown in Fig. 6a. The optimal reaction pH of CapA was 6.0, and the relative activity could be maintained at more than 60% in the range of pH 5.0–6.5. When the pH was less than 5.0 or greater than 6.0, enzyme activity decreased rapidly. Studies on the pH stability showed (Fig. 6b) that CapA was relatively stable at pH 4.0–8.0, and the enzyme activity remained above 60% after 1 h of incubation. CapA was similar to the carboxypeptidases from other sources, which all exhibited the best hydrolysis in acidic conditions. However, unlike the optimal reaction pH of the carboxypeptidases from most filamentous fungi^3,34^ and yeasts^33^, which is near 4.0, the optimal reaction pH and stable pH range of CapA are more neutral. Similar to the reported carboxypeptidase Y from *S. cerevisiae*^25^, CapA plays a role in reaction systems that proceed at a neutral pH, which is more convenient for subsequent product processing after enzyme catalysis has occurred.

#### Influence of metal ions and chemical reagents on enzyme activity

The effects of metal ions or chemical reagents on CapA are shown in Table 2. Mg^2+^ significantly increased the CapA activity, Cu^2+^, Fe^2+^ and CO^2+^ significantly inhibited the CapA activity, and Ca^2+^, Zn^2+^ and low concentrations (1–5 mmol L^−1^) of Mn^2+^ had little effect on the CapA activity, while 10 mmol L^−1^ Mn^2+^ markedly decreased the activity. CapA is a serine protease, and its active center does not need the assistance of metal ions^34^, which is also demonstrated by the fact that the enzyme activity is not affected after ion chelation by EDTA. Therefore, metal ions should form coordination bonds with some of the key amino acids of the enzyme and change its conformation, thus affecting the activity of the enzyme^35^.

**Table 2:** Effect of metal ions or chemicals on the enzymatic activity of CapA.

Metal ions or chemical reagents	Relative activity/%		
	1 mmol L^−1^	5 mmol L^−1^	10 mmol L^−1^
Control	100^Ac^ ± 2.1	100^Ac^ ± 1.3	100^Ab^ ± 1.1
Cu^2+^	20.7^Af^ ± 0.3	9.3^Bf^ ± 0.2	2.7^Ce^ ± 0.1
Mg^2+^	119.4^Ba^ ± 3.6	127.5^Aa^ ± 3.4	108.1^Ca^ ± 2.6
Fe^2+^	42.5^Ae^ ± 1.6	16.4^Be^ ± 1.4	7.5^Cd^ ± 0.7
Ca^2+^	99.4^Ac^ ± 2.1	103.8^Ac^ ± 3.7	98.7^Ab^ ± 1.9
Zn^2+^	97.9^Ac^ ± 1.7	102.5^Ac^ ± 3.3	96.3^Ab^ ± 3.9
Mn^2+^	96.5^Ac^ ± 1.3	90.6^Bd^ ± 2.8	52.1^Cc^ ± 2.2
Co^2+^	76.4^Ad^ ± 1.3	10.4^Bf^ ± 1.2	1.1^Cf^ ± 0.1
Na^2+^	106.4^Ac^ ± 4.2	103.5^Ac^ ± 5.2	104.8^Ab^ ± 3.5
EDTA	107.4^Ab^ ± 2.7	106.5^Ab^ ± 1.8	107.7^Aa^ ± 3.8
PMSF	14.9^Ag^ ± 0.8	/	/

Values are mean ± standard deviation. Analysis performed in triplicate.

Different lowercase superscripts in the same column indicate statistically significant difference (p < 0.05) caused by different metal ions or chemicals within the same concentration. Capital letters in the same row indicate statistically significant difference (p < 0.05) caused by different concentration within the same metal ions or chemicals.

“/” means no detectable enzyme activity.

#### The nature and specificity of CapA

PMSF can inhibit the activity of recombinant enzymes by more than 80%. As a specific inhibitor of serine proteases, PMSF inhibits serine carboxypeptidase (OcpC) and *Monascus* carboxypeptidase^3^. This result clearly demonstrated that CapA is a carboxypeptidase.

CBZ-Pro-Gly, CBZ-Ala-Lys, CBZ-Gly-Ala, CBZ-Ala-Glu and CBZ-Phe-Leu were used as substrates to determine the enzyme kinetic parameters of CapA, and the results are summarized in Table 3. Among the substrates used, the highest affinity (*K*_m_ 0.063 mM) and the highest catalytic efficiency (*k*_cat_/*K*_m_ 186.35 mM^−1^ s^−1^) were both obtained for CBZ-Phe-Leu. CapA has higher specific activity or higher catalytic efficiency than other reported carboxypeptidases^13,36,37^. Therefore, CapA will be more adaptable and less expensive in industrial applications.

**Table 3:** Kinetic parameters for 5 CBZ-aa-aa substrates by carboxypeptidase CapA.

Substrates	*V*_max_ (μmol L^−1^ mg^−1^ s^−1^)	*K*_m_ (mmol L^−1^)	*k*_cat_ (s^−1^)	*k*_cat_*/K*_m_ (mmol L^−1^ s^−1^)
CBZ-Phe-Leu	912.7 ± 93.4	0.063 ± 0.01	11.74 ± 1.02	186.35 ± 16.7
CBZ-Gly-Ala	712.3 ± 83.2	0.24 ± 0.02	1.71 ± 0.08	7.13 ± 0.48
CBZ-Ala-Lys	698.7 ± 74.4	0.18 ± 0.03	2.32 ± 0.17	12.89 ± 1.13
CBZ-Pro-Gly	162.8 ± 14.5	1.17 ± 0.21	0.47 ± 0.02	0.4 ± 0.05
CBZ-Ala-Glu	108.1 ± 11.4	1.44 ± 0.09	0.18 ± 0.01	0.13 ± 0.01

Assays were carried out in 0.1 mol L^−1^ disodium hydrogen phosphate-citric acid buffer, at pH 6.0, 45 °C.

The concentrations of CapA range from 5 to 500 μmol L^−1^.

**Figure 7:**
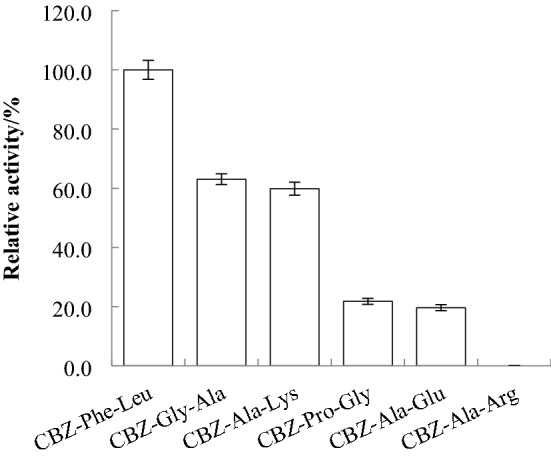
Substrate specificity of recombinant carboxypeptidase.

Using 6 CBZ-AA-AA as substrates, the hydrolysis specificity of CapA was measured and is summarized in Fig. 7. CBZ-Phe-Leu is the optimal substrate for CapA. The ability of CapA to hydrolyze the 6 substrates is as follows: CBZ-Phe-Leu > CBZ-Gly-Ala > CBZ-Ala-Lys > CBZ-Pro-Gly > CBZ-Ala-Glu > CBZ-Ala-Arg. CapA has a wide range of substrate specificities and prefers the carboxy-terminal hydrophobic amino acids Leu and Lys, which cause oligopeptides to be bitter; thus, it has good potential for application in protein C-terminal sequencing and debittering oligopeptides^8,12^. The bitterness of certain peptides is always caused by hydrophobic amino acids. Two carboxypeptidases have been previously used in enzymatic debittering because of their preferred hydrolysis of carboxy-terminal hydrophobic amino acids: a carboxypeptidase from *B. subtilis* completely removed the bitterness of fermented soybean meal and improved the flavor of soybean meal at the same time^38^, and a carboxypeptidase from *Pseudozyma hubeiensis* eliminates the bitterness of bitter peptides generated by proteases^39^. CapA has a similar substrate preference to the abovementioned carboxypeptidases and the advantage of higher specific activity^13,36,37^. Therefore, CapA has the potential to debitter peptides.

There are also reports of using serine carboxypeptidases to accelerate cheese ripening (patent US 20140205717)^40^ and improve the taste of cheese^41^. Recently, a carboxypeptidase extracted from *A. oryzae* was utilized in industry for debittering and improving the flavor of coffee, thereby adding value^42^. The application of CapA in these fields also deserves further investigation.

## Conclusion

In this study, a serine carboxypeptidase CapA from *A niger* was identified for the first time, and its heterologous expression in *P. pastoris* and subsequent purification were successfully carried out. The optimal reaction temperature, temperature stability, optimal reaction pH, pH stability, effects of metal ions and chemical reagents on enzyme activity, kinetic parameters, substrate specificity and other basic enzymatic characteristics of CapA were analyzed. The results show that the optimal reaction temperature and pH of CapA are 45 °C and 6.0, respectively. Compared with the reported carboxypeptidase, CapA has better heat resistance (30% of the enzyme activity is retained after incubation at 60 °C for 2 h) and pH stability (60% of the enzyme activity is retained after incubation at pH 4.0–8.0 for 1 h). Its broad substrate specificity and preference for hydrolyzing carboxy-terminal hydrophobic amino acids indicate that CapA has potential applications in protein C-terminal sequencing and in debittering oligopeptides.
